# Valorization of Garlic (*Allium sativum* L.) Peel Waste Using Natural Deep Eutectic Solvent (NADES) Formulations: Storage Stability and Bioaccessibility of Phenolic Extracts

**DOI:** 10.3390/molecules31142491

**Published:** 2026-07-16

**Authors:** Elif Ceren Kaya, Merve Tomas, Senem Kamiloglu

**Affiliations:** 1 Department of Food Engineering, Faculty of Agriculture, Bursa Uludag University, 16059 Bursa, Türkiye; elifcerenkaya04@gmail.com; 2 Department of Food Engineering, Faculty of Chemical and Metallurgical Engineering, Istanbul Technical University, 34469 Istanbul, Türkiye; mervetomas@itu.edu.tr

**Keywords:** food waste valorization, green extraction, in vitro digestion, antioxidant capacity, flavonoids, phenolic acids

## Abstract

Garlic peel is an underutilized by-product with potential as a source of phenolic compounds. This study evaluated garlic peel waste valorization using ultrasound-assisted extraction with eight natural deep eutectic solvent (NADES) formulations, compared with ethanol and water, in terms of phenolic composition, antioxidant capacity, storage stability, in vitro gastrointestinal behavior, bioaccessibility, and chemometric differentiation. Total phenolic content (TPC), antioxidant capacity, and individual phenolics were analyzed, while extract stability was monitored over 60 days at 4 °C and 25 °C. Glycerol:lactic acid (1:3) and glucose:lactic acid (1:5) exhibited the highest TPC among the NADES formulations, whereas ethanol showed stronger CUPRAC and FRAP responses. HPLC–PDA analysis revealed five flavonoids, including rutin, quercetin, two cyanidin derivatives, and one pelargonidin derivative, and seven phenolic acids, including gallic, protocatechuic, vanillic, chlorogenic, *p*-coumaric, ferulic, and sinapic acids. Selected NADES favored anthocyanin recovery and post-digestion retention of flavonols and hydroxycinnamic acids. Most choline chloride-based NADES enhanced TPC bioaccessibility (105–293%) compared with ethanol and water (86–126%). Storage stability depended on solvent composition and temperature, with 4 °C better preserving phenolic and antioxidant properties. Chemometric analyses confirmed solvent effects on extract composition. NADES-based extraction offers a green strategy for converting garlic peel waste into phenolic-rich functional extracts.

## 1. Introduction

The concept of sustainable nutrition aims to prevent the excessive use of natural resources, promote a more balanced structure in production and consumption processes, and thereby contribute to the establishment of a sustainable food system. In this context, preventing food waste and reducing food losses are among the key elements that constitute the environmental dimension of sustainable nutrition [1]. According to the Food Waste Index Report of the United Nations Environment Programme, approximately 1.05 billion tons of food waste were generated worldwide in 2022, corresponding to nearly 19% of the food available to consumers. Most of the food waste originates from households (60%), followed by the food service sector (28%) and retail sector (12%) [2]. The increasing environmental challenges and economic costs associated with food waste highlight the need to develop next-generation materials and use energy resources more efficiently. In this regard, incorporating food waste into recovery processes, valorizing it through bioconversion, and recovering valuable compounds for reuse are of considerable importance for producing high value-added products [3,4].

Plant extracts have attracted increasing attention in recent years owing to their richness in natural antioxidant compounds and their potential for use in various application areas. In this context, garlic (*Allium sativum* L.), a member of the Amaryllidaceae family, is an important plant source widely used for both medicinal purposes and culinary applications. In addition to imparting a distinctive aroma, taste, and flavor to food products, garlic has also gained considerable attention due to its diverse biological activities [5]. Garlic is believed to have originated in Central Asia approximately 6000 years ago and subsequently spread to Western, Southern, and Eastern Asia, as well as the Mediterranean region [6]. Botanically, garlic is a perennial herbaceous plant that is propagated vegetatively through cloves. Its bulb contains multiple cloves, while the aerial portion bears linear, flattened leaves and, in flowering types, a scape terminating in an umbel [7]. Garlic has long been used as both a food and a traditional remedy, particularly for respiratory and cardiovascular purposes. Commercial preparations include powdered garlic, oil-based liquid extracts, ethanolic dry extracts, and aged garlic extracts, which are commonly formulated as tablets or capsules. Preclinical studies have reported antioxidant, antimicrobial, anti-inflammatory, and cardiometabolic activities, while clinical investigations have mainly focused on the effects of aged garlic extract on blood pressure and other cardiovascular risk markers. However, most clinical evidence concerns bulb-derived preparations [5,6,8]. Garlic peel extracts remain largely at the in vitro or preclinical stage, although their antioxidant and antimicrobial properties indicate potential applications as food ingredients, natural preservatives, nutraceutical ingredients, and active packaging materials [9,10,11,12,13].

Large-scale garlic cultivation and processing generate substantial amounts of agricultural and food-derived by-products. Indeed, it has been reported that garlic processing activities generate more than 3.7 million tons of by-products annually [14]. These by-products may lead to various environmental and economic challenges when they are not properly managed. Nevertheless, they are increasingly recognized as valuable sources of biologically active compounds that can be utilized in the food, health, and agricultural sectors. Garlic peel, which is removed during processing, represents an important by-product that can be valorized as a source of phenolic compounds and other components with antioxidant properties. In this regard, the recovery of phenolic compounds from garlic processing by-products is of considerable importance for converting these materials into high value-added products [15].

Phenolic compounds can be recovered from plant materials not only by conventional methods but also through environmentally friendly extraction techniques. Among these techniques, ultrasound-assisted extraction (UAE) offers several important advantages over conventional extraction methods, including shorter processing times, better preservation of heat-sensitive compounds, and reduced energy consumption [16]. However, UAE also has certain limitations. Its extraction efficiency is strongly influenced by ultrasonic power, frequency, treatment time, temperature, and reactor configuration. Excessive sonication may promote the degradation of sensitive phenolic compounds, while non-uniform cavitation and difficulties in maintaining a consistent acoustic field may complicate process scale-up [17,18]. Despite these limitations, UAE is an effective approach that enhances extraction efficiency by improving the interaction between the plant matrix and the solvent. The cavitation effect generated by ultrasonic waves increases cell wall permeability and facilitates the disruption of plant tissues. As a result, the transfer of phenolic compounds from intracellular structures into the solvent medium is promoted [19]. Organic solvents such as acetone, methanol, ethanol, and isopropanol are commonly mixed with water at different ratios and widely used for the recovery of phenolic compounds from plant sources by UAE [17]. However, the use of these solvents may cause certain environmental concerns and increase the risk of toxic residues in the resulting extracts. Directive 2009/32/EC, which regulates the use of extraction solvents in the production of food and food ingredients, states that food products should not contain solvent residues at levels that may pose a risk to human health [20]. Therefore, new-generation, advanced, and environmentally friendly solvent systems, such as natural deep eutectic solvent (NADES) formulations, have gained increasing importance. NADES formulations are considered promising alternative solvent systems for the green extraction of phenolic compounds due to their low volatility, tunable polarity, biocompatibility, and preparation from naturally occurring components. However, NADES formulations also present several limitations. Their relatively high viscosity can restrict solvent penetration and mass transfer, increase mixing and pumping requirements, and complicate downstream separation and solvent recovery. In addition, their extraction performance and safety are strongly formulation-dependent, and further toxicological, regulatory, and scale-up evaluations are required for many NADES systems [21,22,23]. Nevertheless, recent studies have shown that NADES formulations can exhibit higher extraction performance than conventional organic solvents in the recovery of phenolic compounds from food waste, offering a promising alternative for sustainable extraction processes [24,25]. Nevertheless, the obtained extracts should be evaluated not only in terms of extraction efficiency but also with regard to the stability of their phenolic profile during storage, as this is important for determining their potential application areas.

The beneficial health effects of phenolic compounds are closely associated with their release during gastrointestinal digestion, convertion into absorbable forms, and consequently bioaccessibility. Therefore, evaluating the behavior of phenolic compounds throughout the digestion process is important for better understanding their potential biological effects. In vivo studies are regarded as the most reliable approach for assessing bioavailability, as they more accurately reflect human physiology. However, their applicability is often limited by ethical constraints, high costs, and methodological challenges. In this context, in vitro digestion models are widely used to simulate gastrointestinal conditions, as they provide rapid results, ensure reproducibility, and offer a more ethically feasible alternative [26]. The standardized INFOGEST in vitro digestion model was developed for this purpose and enables the physiologically relevant and reproducible simulation of gastrointestinal conditions [27,28]. Nevertheless, studies on the in vitro bioaccessibility of phenolic compounds obtained using NADES formulations remain limited [29,30,31,32,33].

Previous studies have demonstrated the potential of garlic processing by-products as sources of phenolic compounds and antioxidants. However, most investigations have employed aqueous or conventional organic solvents, and have primarily focused on extraction yield, total phenolic content, phenolic composition, or antioxidant activity [9,10,11]. NADES formulations have also been successfully applied to the recovery of phenolic compounds from several agri-food by-products, including citrus peels, walnut shells, sunflower seed shells, and quince by-products, demonstrating that extraction performance, stability, and gastrointestinal behavior are dependent on both the plant matrix and solvent composition [30,31,33,34]. Although NADES formulations have recently been investigated for the recovery of bioactive compounds from black garlic peel, the resulting extracts were evaluated mainly for their cosmeceutical potential rather than their storage stability and gastrointestinal bioaccessibility [35]. Therefore, to the best of our knowledge, the present study is the first to systematically compare NADES formulations with ethanol and water for the UAE-based recovery of phenolic compounds from raw garlic peel waste through an integrated assessment of individual phenolic composition, antioxidant capacity, 60-day storage stability, in vitro bioaccessibility, and chemometric differentiation.

Accordingly, this study aimed to recover phenolic compounds from garlic peel waste by combining NADES with UAE and to compare the resulting extracts with those obtained using ethanol and water. The phenolic composition and antioxidant capacity of the extracts were evaluated together with their stability during 60 days of storage at 4 °C and 25 °C. In addition, their behavior during standardized in vitro gastrointestinal digestion and the bioaccessibility of their phenolic compounds were investigated. Correlation analysis, principal component analysis (PCA), and hierarchical cluster analysis (HCA) were also applied to determine the relationships among the extraction solvents, phenolic profiles, and antioxidant properties.

## 2. Results and Discussion

### 2.1. Characterization of NADES Formulations

The prepared NADES formulations were characterized in terms of density, pH, and FTIR spectra to evaluate their physicochemical properties and obtain information on their functional-group profiles and possible intermolecular interactions. The density of the NADES formulations ranged from 1.1169 g/cm^3^ for choline chloride:lactic acid (1:2) (NADES 4) to 1.3902 g/cm^3^ for choline chloride:glycerol (1:2) (NADES 6), whereas their pH values varied between 0.45 for choline chloride:citric acid (1:2) (NADES 5) and 4.72 for choline chloride:xylose (1:2) (NADES 2) (Table 1). The density values are consistent with the literature, which indicates that most NADES formulations exhibit higher densities than water (1 g/cm^3^) and ethanol, a commonly used organic solvent with a density below 1 g/cm^3^ [36]. The pH of NADES formulations may influence the extraction efficiency of phenolic compounds by affecting their ionization state, stability, and interactions with the solvent system. Acidic NADES formulations can favor phenolic extraction by improving solubilization and promoting hydrogen-bonding interactions between phenolics and the solvent components, although this effect also depends on NADES composition and other physicochemical properties [37].

Viscosity is an important physicochemical property of NADES formulations because high viscosity can limit solvent–matrix mass transfer and thereby influence extraction performance [38]. Although the addition of 30% water was expected to reduce the viscosity of the prepared systems, differences among the formulations may still have contributed to the observed variation in phenolic extraction. However, viscosity was not experimentally measured in the present study, which represents a limitation when interpreting formulation-dependent differences in extraction performance. Therefore, the possible contribution of viscosity and mass transfer to the observed results could not be quantitatively established and should be investigated in future studies.

FTIR analysis was performed to characterize the functional groups present in the prepared NADES formulations, and the corresponding spectra are presented in Supplementary Material Figure S1. All formulations exhibited a broad absorption band in the 3500–3000 cm^−1^ region, corresponding mainly to overlapping O–H stretching vibrations from the hydroxyl-containing solvent components. The broad nature of this band is consistent with the presence of different hydrogen-bonding environments within the solvent systems. Weak absorption bands in the 3000–2850 cm^−1^ region were attributed to aliphatic C–H stretching vibrations. The organic acid-containing formulations (NADES formulations 3–5, 7, and 8) displayed pronounced absorption in the approximately 1750–1680 cm^−1^ region associated with C=O stretching of carboxylic acid groups, whereas this feature was less evident in other NADES formulations. In the fingerprint region, bands at approximately 1450–1350 cm^−1^ were associated mainly with C–H bending, while the intense absorptions between 1200 and 900 cm^−1^ were attributed to C–O and C–O–C stretching vibrations, particularly in the glucose-, xylose-, and glycerol-containing formulations [39,40]. The differences observed in the carbonyl and fingerprint regions were consistent with the different chemical structures of the NADES components. The spectra were consistent with hydrogen-bonded environments within the prepared NADES formulations [40]. However, because interactions between the NADES components and the extracted phenolic compounds were not directly investigated, the FTIR results should not be interpreted as evidence of compound-specific extraction mechanisms.

### 2.2. Total Phenolic Content

**Figure 1:**
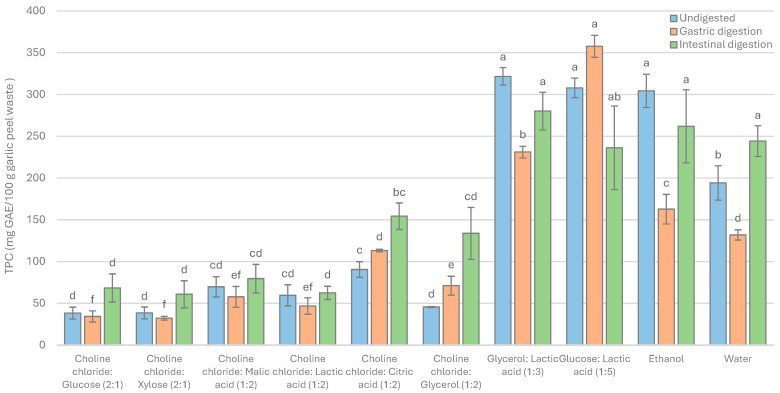
Changes in the total phenolic content (TPC) of garlic peel waste extracts during in vitro digestion. Results are expressed as the mean ± standard deviation of three independent measurements. Different letters above the bars indicate statistically significant differences among samples, as determined by one-way ANOVA followed by Tukey’s test (*p* < 0.05). GAE: gallic acid equivalents.

Garlic peel waste extracts were obtained using UAE with eight different NADES formulations, and their TPC was compared with those of ethanol and water extracts (Figure 1). Before digestion, the highest TPC was observed in the glycerol:lactic acid (1:3) (NADES 7) and glucose:lactic acid (1:5) (NADES 8) extracts. Although the TPC levels of these extracts were higher than that of the ethanol extract, the difference was not statistically significant (*p* > 0.05). In contrast, the water extract exhibited a significantly higher TPC than the choline chloride-based NADES extracts (*p* < 0.05). These findings demonstrate that NADES should not be considered a uniform solvent class, as their extraction performance depended strongly on the specific solvent composition under the applied conditions. The contrasting TPC responses of NADES 7 and NADES 8 and the choline chloride-based formulations further demonstrate that extraction performance was formulation-dependent. However, because solvent polarity, viscosity, mass transfer, and solvent–phenolic interactions were not directly evaluated, the physicochemical basis of these differences cannot be established from the present results. A similar formulation-dependent pattern has been reported for onion peel, another Allium by-product, where choline chloride-based deep eutectic solvents prepared with different hydrogen-bond donors showed markedly different polyphenol extraction efficiencies [41]. This comparison further supports the interpretation that extraction performance depends on the specific solvent composition rather than on the use of a eutectic solvent alone.

Changes in the TPC of the garlic peel waste extracts during the gastric and intestinal phases of digestion are presented in Figure 1. After gastric digestion, the glycerol:lactic acid (1:3) (NADES 7) and NADES 8 extracts exhibited significantly higher TPC than the ethanol and water extracts (*p* < 0.05). Following intestinal digestion, the glycerol:lactic acid (1:3) (NADES 7) extract continued to show higher TPC than the ethanol and water extracts, although this difference was not statistically significant (*p* > 0.05). Intestinal digestion resulted in an increase in the TPC of the extracts compared with their undigested counterparts, with this increase being more pronounced in choline chloride-based extracts. This increase may reflect digestion-induced changes in the extract matrix and in the accessibility or reducing reactivity of phenolic and non-phenolic constituents. However, the mechanism underlying this increase cannot be determined from these data alone.

When the bioaccessibility results were evaluated, most choline chloride-based extracts showed higher apparent TPC bioaccessibility values (105–293%) than the ethanol and water extracts (86–126%). Among these extracts, choline chloride:glycerol (1:2) (NADES 6) exhibited the highest bioaccessibility index. The different rankings observed for initial TPC and post-digestion bioaccessibility further indicate that the performance of each NADES formulation depends on the specific outcome being evaluated. The high bioaccessibility observed for NADES 6 may be associated with its glycerol-containing composition. Previous studies have reported that glycerol-based NADES may facilitate the release of bound phenolics during digestion and improve their bioaccessibility [33]. However, the molecular interactions responsible for this effect were not directly investigated in the present study. Conversely, the relatively low bioaccessibility values of glycerol:lactic acid (1:3) (NADES 7) and NADES 8 could reflect limited phenolic release during digestion or possible compound–solvent and compound–matrix interactions affecting post-digestion quantification. Since these interactions were not characterized, this interpretation remains tentative.

Bioaccessibility values above 100% should not be interpreted as indicating that a greater absolute amount of phenolic compounds was recovered after digestion than was initially present. Because the bioaccessibility index was calculated from the Folin–Ciocalteu responses measured before and after digestion, values above 100% indicate that the digested samples produced a higher assay response than the corresponding undigested samples. This may reflect digestion-induced changes in the extract matrix, increased accessibility of reducing constituents, or the formation of transformation products with different reducing properties. Although corresponding solvent blanks were used to minimize direct contributions from the NADES matrices, the Folin–Ciocalteu assay is not selective for phenolic compounds and may respond to other reducing constituents, including sugars and organic acids [42]. Therefore, these values should be interpreted as apparent TPC bioaccessibility rather than as quantitative recovery of phenolic compounds. In the present study, TPC was consequently used as a broad comparative measure, whereas compound-specific conclusions regarding extraction and digestion behavior were based primarily on the targeted HPLC–PDA data.

**Figure 2:**
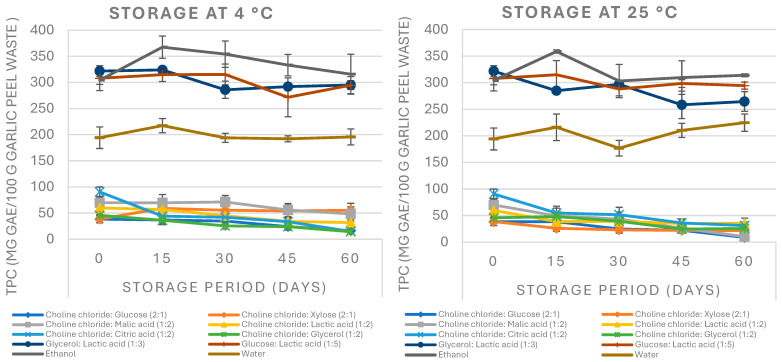
Changes in the total phenolic content (TPC) of garlic peel waste extracts during storage at 4 °C and 25 °C. Results are expressed as the mean ± standard deviation of three independent measurements. GAE: gallic acid equivalents.

To evaluate the storage stability of the NADES extracts, they were stored at 4 °C and 25 °C for 60 days, and their TPC was determined at 15-day intervals (Figure 2). TPC remained largely stable in the NADES 8, ethanol, and water extracts throughout storage at both 4 °C and 25 °C, whereas most NADES extracts exhibited time- and temperature-dependent reductions. These decreases occurred earlier and were more pronounced in choline chloride-based systems stored at 25 °C, with most changes becoming evident between days 15 and 30 of storage. In comparison, storage at 4 °C delayed these reductions. Some extracts, such as choline chloride:xylose (2:1) (NADES 2) and choline chloride:malic acid (1:2) (NADES 3), maintained their stability only under refrigerated conditions. These findings are consistent with a previous study on NADES extracts obtained from orange peel, which reported that TPC values during storage vary depending on solvent composition and temperature, and that low-temperature storage can support the preservation of phenolic compounds [34].

During storage, phenolic compounds may undergo several transformation reactions, including oxidation to quinone-type products and subsequent coupling, condensation, or polymerization reactions [43]. These transformations may decrease the concentration of the original phenolic compounds or generate products with different reducing properties. Since the Folin–Ciocalteu assay primarily reflects the reducing capacity of a sample and its response depends on phenolic structure, such transformations may result in changes in the measured TPC response [44]. The rate and extent of phenolic transformation can also be influenced by pH and oxygen exposure [45]. Therefore, the earlier reductions observed in several extracts at 25 °C may partly reflect accelerated chemical degradation, whereas differences among the extracts may be associated with the distinct chemical environments provided by the solvent systems. However, individual degradation products and specific phenolic compounds were not monitored by HPLC–PDA during storage. Therefore, the proposed degradation pathways remain tentative, and the observed changes in TPC should be interpreted as changes in the overall Folin–Ciocalteu response rather than as direct evidence of the degradation or preservation of specific phenolic compounds.

### 2.3. Total Antioxidant Capacity

**Figure 3:**
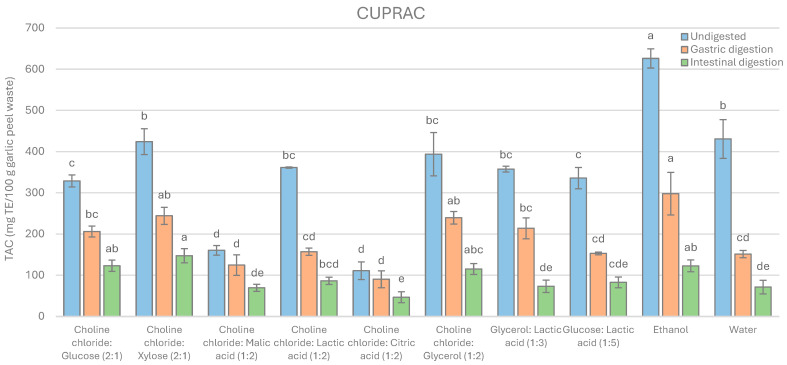
Changes in the total antioxidant capacity (TAC) of garlic peel waste extracts during in vitro digestion, based on reducing capacity measured by the CUPRAC assay. Results are expressed as the mean ± standard deviation of three independent measurements. Different letters above the bars indicate statistically significant differences among samples, as determined by one-way ANOVA followed by Tukey’s test (*p* < 0.05). TE: Trolox^®^ equivalents.

**Figure 4:**
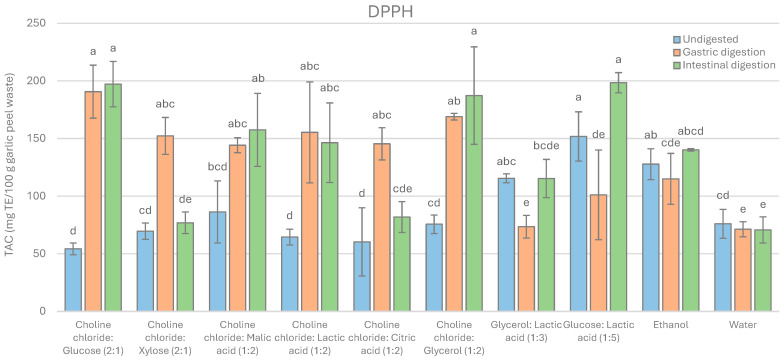
Changes in the total antioxidant capacity (TAC) of garlic peel waste extracts during in vitro digestion, based on radical-scavenging capacity measured by the DPPH assay. Results are expressed as the mean ± standard deviation of three independent measurements. Different letters above the bars indicate statistically significant differences among samples, as determined by one-way ANOVA followed by Tukey’s test (*p* < 0.05). TE: Trolox^®^ equivalents.

**Figure 5:**
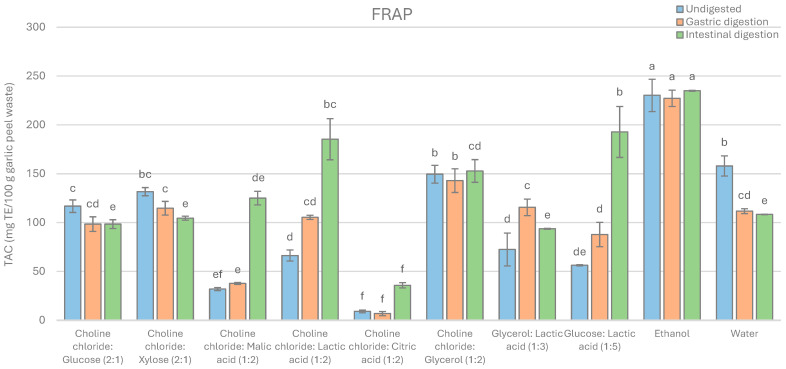
Changes in the total antioxidant capacity (TAC) of garlic peel waste extracts during in vitro digestion, based on reducing capacity measured by the FRAP assay. Results are expressed as the mean ± standard deviation of three independent measurements. Different letters above the bars indicate statistically significant differences among samples, as determined by one-way ANOVA followed by Tukey’s test (*p* < 0.05). TE: Trolox^®^ equivalents.

The TAC of garlic peel waste extracts obtained using UAE with eight different NADES formulations was evaluated using three assays (CUPRAC, DPPH, and FRAP) and compared with those of ethanol and water extracts (Figure 3, Figure 4 and Figure 5). Before digestion, the CUPRAC and FRAP assays showed that the ethanol extract exhibited significantly higher reducing capacity than all NADES and water extracts (*p* < 0.05). On the other hand, according to the DPPH assay, the glucose:lactic acid (1:5) (NADES 8) extract showed the highest radical-scavenging capacity before digestion, followed by the ethanol and glycerol:lactic acid (1:3) (NADES 7) extracts, with no statistically significant differences among them (*p* > 0.05). The differences among the CUPRAC, DPPH and FRAP assays may be attributed to their distinct reaction mechanisms. CUPRAC and FRAP mainly reflect the reducing capacity of antioxidants through electron-transfer reactions, whereas DPPH evaluates radical-scavenging ability [42]. Accordingly, the higher reducing capacity of the ethanol extract in the CUPRAC and FRAP assays suggests the presence of compounds with stronger reducing capacity, while the higher DPPH response of NADES 8 may indicate greater radical-scavenging capacity. These findings demonstrate that the ranking of the solvent systems was assay-dependent. Therefore, CUPRAC, DPPH, and FRAP should be interpreted as method-specific measures of reducing or radical-scavenging reactivity rather than as interchangeable measurements of a single antioxidant property. Accordingly, no solvent system can be considered broadly superior across all antioxidant endpoints.

Changes in the TAC of the garlic peel waste extracts during the gastric and intestinal phases of digestion are presented in Figure 3, Figure 4 and Figure 5. Following gastric digestion, CUPRAC assay results showed that the ethanol extract exhibited the highest reducing capacity, followed by NADES 6 and choline chloride:xylose (2:1) (NADES 2), although the differences among these latter extracts were not statistically significant (*p* > 0.05) (Figure 3). According to the DPPH assay, choline chloride:glucose (2:1) (NADES 1) showed the highest radical-scavenging capacity after gastric digestion (Figure 4). In contrast, the FRAP assay indicated that the ethanol extract had significantly higher reducing capacity than all NADES and water extracts after gastric digestion (Figure 5).

After intestinal digestion, the CUPRAC assay revealed that NADES 2 had the highest TAC, followed by NADES 1, ethanol, and NADES 6, with no statistically significant differences among these extracts (*p* > 0.05) (Figure 3). Based on the DPPH assay, NADES 8, NADES 1, and NADES 6 displayed the highest TAC values after intestinal digestion, again without statistically significant differences (*p* > 0.05) (Figure 4). Similar to the gastric phase, the FRAP assay showed that the ethanol extract had significantly higher TAC than all NADES and water extracts after intestinal digestion (*p* < 0.05) (Figure 5).

After in vitro digestion, assay-dependent changes were observed in the TAC of the extracts. Reducing capacity decreased according to the CUPRAC assay, whereas radical-scavenging capacity increased according to the DPPH assay. The FRAP reducing capacity showed a variable trend, with both increases and decreases depending on the extract type. These differences may be partly explained by the broader applicability of the CUPRAC assay to both hydrophilic and lipophilic antioxidants, whereas the DPPH assay is more responsive to antioxidants soluble in organic media, particularly those with relatively hydrophobic characteristics [42]. Additionally, non-phenolic compounds may also have contributed to the overall antioxidant potential.

The apparent bioaccessibility of TAC varied depending on the antioxidant capacity assay used. According to the CUPRAC assay, all NADES extracts (20–43%) showed higher bioaccessibility than the ethanol and water extracts (17–20%). For the DPPH and FRAP assays, most NADES extracts (79–392%) also exhibited higher bioaccessibility than the conventional solvent extracts (69–110%), although this trend was not observed for all samples. Based on the CUPRAC and FRAP assays, NADES 3 and choline chloride:citric acid (1:2) (NADES 5) had the highest bioaccessibility values, whereas NADES 1 showed the highest bioaccessibility according to the DPPH assay. The higher bioaccessibility observed for NADES 3 and NADES 5 may be partly related to their relatively low pH values (Table 1), which could promote the stability of antioxidant compounds under digestion conditions. Moreover, interactions between the solvent systems and assay reagents may have influenced the measured antioxidant activity, potentially explaining the variations in NADES rankings across the different methods. Values above 100% should not be interpreted as a literal increase in the amount of antioxidant compounds. Since these indices were calculated from antioxidant assay responses before and after digestion, values above 100% indicate increased post-digestion reactivity in the corresponding assay. Changes in pH, exposure to digestive enzymes and bile salts, alterations in matrix composition, and the formation of compounds with different electron-donating or radical-scavenging properties may all influence the measured response. Moreover, because CUPRAC, DPPH, and FRAP are based on different reaction mechanisms, the same digested extract may produce substantially different apparent bioaccessibility values. These results should therefore be interpreted as assay-dependent changes in antioxidant reactivity rather than as quantitative recovery of antioxidant compounds.

**Figure 6:**
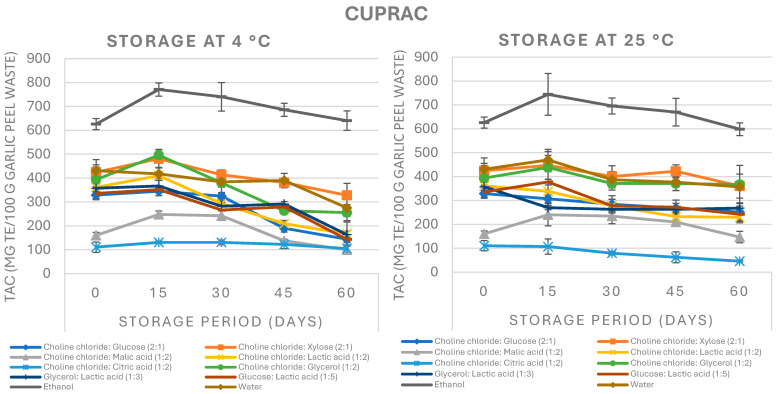
Changes in the total antioxidant capacity (TAC) of garlic peel waste extracts, as determined by the CUPRAC assay, during storage at 4 °C and 25 °C. Results are expressed as the mean ± standard deviation of three independent measurements. TE: Trolox^®^ equivalents.

**Figure 7:**
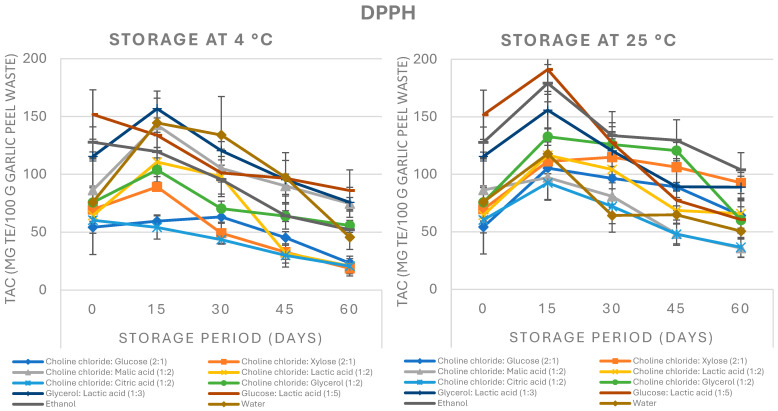
Changes in the total antioxidant capacity (TAC) of garlic peel waste extracts, as determined by the DPPH assay, during storage at 4 °C and 25 °C. Results are expressed as the mean ± standard deviation of three independent measurements. TE: Trolox^®^ equivalents.

**Figure 8:**
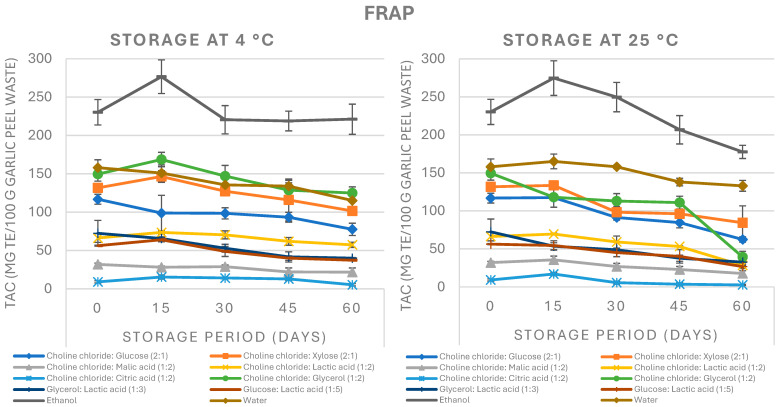
Changes in the total antioxidant capacity (TAC) of garlic peel waste extracts, as determined by the FRAP assay, during storage at 4 °C and 25 °C. Results are expressed as the mean ± standard deviation of three independent measurements. TE: Trolox^®^ equivalents.

The storage stability of the NADES extracts was assessed over 60 days at 4 °C and 25 °C by monitoring TAC at 15-day intervals using the CUPRAC, DPPH, and FRAP assays (Figure 6, Figure 7 and Figure 8). According to the CUPRAC assay, an initial increase in CUPRAC reducing capacity was observed after 15 days of storage in the choline chloride:malic acid (1:2) (NADES 3), choline chloride:glycerol (1:2) (NADES 6), and ethanol extracts. This transient increase should be regarded as an observed change in the CUPRAC response during storage. Possible contributors may include changes in compound accessibility or the formation of transformation products with altered reducing properties. Similarly, the subsequent decline could potentially be associated with oxidation, condensation, polymerization, or other chemical transformations. However, because the compounds and transformation products responsible for these changes were not identified, these mechanisms cannot be confirmed and are presented only as possible explanations. In the remaining extracts, CUPRAC reducing capacity decreased over time, with the extent of reduction depending on solvent composition and storage temperature (Figure 6). A similar pattern was observed for the DPPH radical-scavenging assay, where several extracts showed a transient increase after 15 days, followed by a gradual decrease. More pronounced reductions were observed at 4 °C, whereas at 25 °C, the onset of these decreases was delayed (Figure 7). In contrast, FRAP reducing capacity indicated greater stability at 4 °C, with no significant changes in several NADES and ethanol extracts, while the water extract showed reductions only after prolonged storage. Storage at 25 °C resulted in earlier declines in multiple systems, with some reductions appearing as early as day 15, whereas changes in the ethanol and water extracts became evident only at the end of storage (Figure 8). These results suggest that the retention of TAC during storage was not uniform across the extracts, but depended on both the NADES formulation and the antioxidant mechanism measured by each assay. This is consistent with previous reports indicating that the storage stability of NADES-based extracts is strongly influenced by solvent composition and storage conditions [22,34].

Considering initial extraction performance together with storage behavior, NADES 8 showed the most favorable balance among the NADES formulations. It exhibited one of the highest initial Folin–Ciocalteu responses, the highest initial DPPH response, and comparatively stable TPC throughout 60 days of storage at both temperatures. NADES 7 also showed strong initial TPC and DPPH responses, but its storage stability was less consistent, suggesting that additional stabilization or shorter storage periods may be required. The choline chloride-based formulations showed lower initial TPC and more pronounced time- and temperature-dependent decreases, particularly at 25 °C. NADES 2 and NADES 3 retained TPC more effectively under refrigerated conditions, indicating that their practical application may require cold storage. However, these comparisons remain endpoint-specific because storage behavior differed among the CUPRAC, DPPH, and FRAP assays, and compound-specific phenolic stability was not evaluated by HPLC–PDA during storage.

### 2.4. Flavonoids

The HPLC–PDA analysis of garlic peel waste extracts enabled the identification of five major flavonoid compounds, including two flavonols and three anthocyanins (Table 2). The identified flavonols were rutin and quercetin, while the anthocyanins were tentatively assigned as two cyanidin derivatives and one pelargonidin derivative based on their UV–Vis spectral characteristics. The identification of these compounds was in line with previous reports in the literature [46,47,48,49].

In the undigested extracts, the ethanol extract exhibited higher flavonol levels than the NADES and water extracts. However, no statistically significant difference was observed between the ethanol extract and glycerol:lactic acid (1:3) (NADES 7) in terms of rutin content (*p* > 0.05). When the total flavonol content was considered, the ethanol extract showed significantly higher levels than all other extracts (*p* < 0.05). The higher total flavonol content of the ethanol extract may be related to the solubility behavior of rutin and quercetin in an intermediate-polarity solvent. However, the polarity of the solvents and the solubility of these individual flavonols were not directly evaluated. Previous studies have similarly reported that ethanol-based solvents may provide comparable or higher flavonoid extraction efficiency than some NADES formulations, depending on solvent composition and the target compounds [50]. Furthermore, all NADES extracts exhibited higher flavonol contents than the water extract, with the exception of choline chloride:citric acid (1:2) (NADES 5). This result may be related to the greater solubility of garlic peel waste flavonols in solvents with lower polarity than water [51].

Regarding the anthocyanin profile of the undigested extracts, anthocyanins were not detected in the water extract, indicating the limited ability of water to recover these compounds under the applied extraction conditions. In contrast, several NADES extracts, particularly choline chloride:malic acid (1:2) (NADES 3), choline chloride:lactic acid (1:2) (NADES 4), choline chloride:glycerol (1:2) (NADES 6), and glucose:lactic acid (1:5) (NADES 8), exhibited higher total anthocyanin contents than the ethanol extract (*p* < 0.05). The higher anthocyanin contents observed in several NADES extracts compared with the ethanol extract are consistent with previous reports indicating that NADES can be highly effective extraction media for anthocyanins. For instance, NADES-based systems have been reported to improve anthocyanin recovery from mulberry [52] and grape pomace [53] compared with conventional organic solvents including ethanol-based systems. Previous studies have associated enhanced anthocyanin recovery by NADES extracts with formulation-dependent polarity and possible stabilization within hydrogen-bonded solvent networks [52,53]. However, these mechanisms were not directly investigated in the present study.

After in vitro gastrointestinal digestion, anthocyanins were not detected in any of the extracts, suggesting that these compounds were highly susceptible to degradation under the applied digestion conditions. Recent studies have similarly reported substantial reductions in anthocyanin recovery during the intestinal phase of simulated digestion. The extent of this reduction was influenced by anthocyanin structure and the surrounding food matrix, indicating that the transition from acidic gastric conditions to the intestinal environment may be particularly important for anthocyanin stability [54,55]. In terms of flavonols, rutin was not detected in the digested NADES 5, ethanol, or water extracts, which may be associated with deglycosylation, hydrolysis, and/or degradation reactions occurring during gastrointestinal digestion, particularly under intestinal conditions. When the total flavonol content after digestion was considered, NADES 3, NADES 4, NADES 7, and NADES 8 showed significantly higher levels than both the ethanol and water extracts (*p* < 0.05). Moreover, all NADES extracts exhibited higher flavonol bioaccessibility (25–75%) than the ethanol extract (16%), while several NADES extracts also showed higher bioaccessibility than the water extract (40%). Among them, choline chloride:glucose (2:1) (NADES 1), choline chloride:xylose (2:1) (NADES 2), and NADES 3 were the most prominent extracts, with flavonol bioaccessibility values exceeding 70%. The higher flavonol bioaccessibility observed in certain NADES extracts is consistent with previous studies reporting that NADES may improve the solubility, stability, and bioaccessibility of rutin and other flavonols [56,57]. Hydrogen-bonding interactions between flavonols and NADES constituents have been proposed as one possible explanation for these effects. However, such interactions and their influence on flavonol solubility, precipitation, and degradation were not directly investigated in the present study.

### 2.5. Phenolic Acids

The HPLC–PDA analysis of garlic peel waste extracts enabled the identification of seven major phenolic acids, including three hydroxybenzoic acids and four hydroxycinnamic acids (Table 3). The identified hydroxybenzoic acids were gallic, protocatechuic, and vanillic acids, whereas chlorogenic, *p*-coumaric, ferulic, and sinapic acids were assigned as hydroxycinnamic acids. The identification of these compounds was in line with previous reports in the literature [46,48,49,58,59].

In the undigested extracts, no hydroxybenzoic acid derivatives were detected in choline chloride:citric acid (1:2) (NADES 5). Similarly, protocatechuic acid and vanillic acid were not detected in several NADES extracts or in the water extract, indicating that the recovery of individual hydroxybenzoic acids may be influenced by solvent composition and the selectivity of the extraction system. With the exception of choline chloride:malic acid (1:2) (NADES 3) and choline chloride:lactic acid (NADES 4), the total hydroxybenzoic acid contents of the NADES extracts did not differ significantly from those of the ethanol and water extracts (*p* > 0.05). Notably, choline chloride:glycerol (NADES 6) exhibited the highest hydroxybenzoic acid content, suggesting that this solvent system may be more favorable for the recovery of hydroxybenzoic acid derivatives.

A different trend was observed for hydroxycinnamic acids in the undigested extracts. The ethanol extract had a significantly higher total hydroxycinnamic acid content than all NADES extracts (*p* < 0.05). In contrast, all NADES extracts, except NADES 5, showed higher hydroxycinnamic acid contents than the water extract. These findings indicate that ethanol was more effective in extracting hydroxycinnamic acids, while most NADES formulations still performed better than water for this phenolic acid subclass. The differences observed between hydroxybenzoic and hydroxycinnamic acids suggest that the extraction efficiency of NADES may depend not only on solvent composition but also on the structural characteristics and polarity of the target phenolic acids.

After in vitro gastrointestinal digestion, the detectability of hydroxybenzoic acids decreased markedly. Among these compounds, gallic acid was detected only in the glycerol:lactic acid (1:3) (NADES 7) and glucose:lactic acid (1:5) (NADES 8) extracts, while no other hydroxybenzoic acid derivatives were detected in the digested samples. The absence of these compounds after digestion may be associated with their degradation or reduced solubility under gastrointestinal conditions, particularly during the intestinal phase. Their concentrations may also have decreased below the detection limit due to pH-dependent instability, oxidation, precipitation, or interactions with the digestive matrix [60].

For hydroxycinnamic acids, sinapic acid was not detected in any of the digested extracts, and some other derivatives were also absent in certain samples after digestion. This indicates that hydroxycinnamic acids may exhibit compound- and solvent-dependent stability patterns during gastrointestinal digestion. Among the digested samples, NADES 4 and NADES 8 exhibited significantly higher total hydroxycinnamic acid contents than both the ethanol and water extracts (*p* < 0.05). These results suggest that certain NADES formulations may provide a more favorable environment for the retention or release of hydroxycinnamic acid derivatives during gastrointestinal digestion.

Recent evidence confirms that gastrointestinal behavior is highly compound- and matrix-dependent. Li et al. [61] reported structure-dependent differences in the digestive stability of individual phenolic compounds, with greater degradation of several compounds during the intestinal phase. In purple rice bran, gallic, protocatechuic, vanillic, *p*-coumaric, ferulic, and sinapic acids also showed different stability and bioaccessibility patterns [55]. Conversely, increases in free ferulic and *p*-coumaric acids during intestinal digestion have been reported in rice matrices, suggesting that digestion may also promote the release of matrix-bound phenolic acids [62].

The bioaccessibility of phenolic acids after in vitro gastrointestinal digestion was also evaluated. Since hydroxybenzoic acids were detected only in the NADES 7 and NADES 8 extracts after digestion, the bioaccessibility of this phenolic acid subclass was calculated only for these two extracts, both of which showed a bioaccessibility value of 22%. In terms of hydroxycinnamic acids, NADES 7 exhibited the highest total hydroxycinnamic acid bioaccessibility (69%), exceeding those of the ethanol, water, and other NADES extracts (9–66%). This finding suggests that the glycerol:lactic acid (1:3) system may be particularly effective in maintaining or releasing hydroxycinnamic acid derivatives during gastrointestinal digestion. A previous study using the same NADES system reported a chlorogenic acid bioaccessibility of 61% in sunflower seed shell extracts [31], which is comparable to the value obtained for total hydroxycinnamic acids in the present study.

Importantly, the solvent rankings obtained using the Folin–Ciocalteu assay did not fully align with those derived from the targeted HPLC–PDA analysis. Although NADES 7 and NADES 8 exhibited the highest TPC responses among the NADES extracts, ethanol contained the highest levels of total flavonols and hydroxycinnamic acids, whereas selected NADES formulations showed advantages in anthocyanin recovery and the post-digestion retention of flavonols and hydroxycinnamic acids. This divergence further indicates that the Folin–Ciocalteu response should be considered a broad comparative measure rather than a direct measure of total phenolic concentration. Accordingly, greater interpretive weight was given to the compound-specific HPLC–PDA results. Consequently, comparisons of solvent performance in the present study were restricted to the specific assay response or phenolic subclass being evaluated.

It should be emphasized that the static INFOGEST model estimates the fraction of phenolic compounds released from the matrix and potentially available for intestinal absorption under simulated gastrointestinal conditions. However, it does not account for intestinal epithelial uptake, first-pass metabolism, systemic distribution, colonic microbial transformations, or interindividual physiological variability [28]. Therefore, the bioaccessibility values obtained in the present study should not be interpreted as direct evidence of in vivo bioavailability or biological efficacy. Cell-based absorption models and subsequent in vivo studies are required to confirm the absorption, metabolism, and physiological effects of the recovered phenolic compounds.

### 2.6. Correlation Analysis, Principal Component Analysis (PCA), and Hierarchical Cluster Analysis (HCA)

Pearson correlation analysis (Supplementary Material Figure S2) revealed a strong positive relationship between TPC and DPPH activity (*r* = 0.88), whereas its correlations with CUPRAC and FRAP were weaker (*r* ≤ 0.40). This finding indicates that the contribution of phenolic compounds to antioxidant capacity may vary depending on the reaction mechanism of the assay used. The observed correlations represent statistical associations among distinct analytical endpoints and should not be interpreted as indicating that TPC, CUPRAC, DPPH, FRAP, and HPLC–PDA measure the same chemical phenomenon. The strong positive correlation between CUPRAC and FRAP (*r* = 0.92) suggests that these two assays reflected similar reducing capacity trends. The moderate-to-strong positive correlations of TPC with rutin, quercetin, gallic acid, and ferulic acid (*r* ≥ 0.62) indicate that these compounds may be among the main contributors to the TPC of garlic peel extracts. In addition, the strong positive correlations observed among rutin, quercetin, chlorogenic acid, and ferulic acid (*r* ≥ 0.69) suggest that these compounds may have been co-extracted under similar extraction conditions or similarly retained within the extracts. The high correlations among anthocyanin derivatives (*r* ≥ 0.72) further indicate that these compounds exhibited similar distribution patterns and behavior across the extracts. However, the negative or weak correlations of some phenolic acids, such as protocatechuic and vanillic acids, with TPC and DPPH activity should not be interpreted as an absence of contribution to antioxidant activity. Indeed, their positive correlations, particularly with FRAP (*r* ≥ 0.63), support the view that antioxidant response may vary depending on both compound structure and the assay employed. These findings suggest that the antioxidant capacity of garlic peel waste extracts was not determined by a single phenolic group, but rather by the overall phenolic profile, the distribution of individual compounds, and the reaction mechanism of each antioxidant assay.

**Figure 9:**
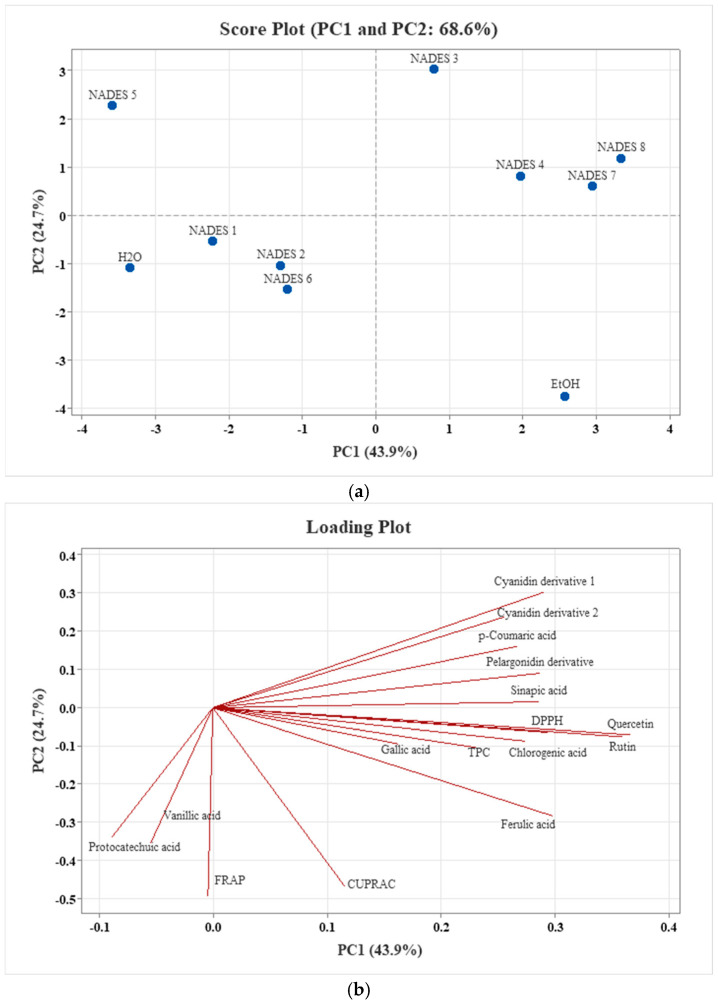
Principal component analysis (PCA) plots showing the relationships between the phenolic composition and antioxidant properties of garlic peel waste extracts. (**a**) Score plot, (**b**) loading plot.

The PCA score and loading plots, showing the distribution of garlic peel waste extracts and the contribution of variables to the principal components, are presented in Figure 9. Based on the eigenvalue > 1.00 criterion, three principal components were retained and together explained 85.7% of the total variance (Supplementary Material, Table S1). The first two components accounted for 68.6% of the variance, with PC1 and PC2 explaining 43.9% and 24.7%, respectively. PC1 was mainly associated with the flavonols quercetin and rutin, which showed the highest positive loadings. Positive loadings were also observed for the hydroxycinnamic acids ferulic, sinapic, chlorogenic, and *p*-coumaric acids, the anthocyanins cyanidin derivative 1 and the pelargonidin derivative, and DPPH activity. Therefore, the positive side of PC1 can be interpreted as representing extracts more strongly associated with these phenolic groups and with DPPH activity. The score plot showed a clear separation among the extracts depending on the extraction solvent. Glycerol:lactic acid (1:3) (NADES 7) and glucose:lactic acid (1:5) (NADES 8) were distinctly positioned on the positive side of PC1, indicating their strong association with the major phenolic compounds contributing to this component. Choline chloride:lactic acid (NADES 4) was also located in the positive PC1 region, suggesting a relatively similar phenolic profile, although less pronounced than NADES 7 and NADES 8. These results indicate that lactic acid-containing NADES formulations were particularly effective in obtaining phenolic-rich garlic peel waste extracts. Ethanol was also positioned on the positive side of PC1, confirming its effectiveness in extracting phenolic compounds. However, it was clearly separated from NADES 7 and NADES 8 along PC2 and located in the negative PC2 region, which was mainly characterized by strong negative loadings of FRAP and CUPRAC. This suggests that ethanol was more closely associated with reducing capacity-based antioxidant responses, whereas NADES 7 and NADES 8 were more strongly associated with the flavonols, anthocyanins, and hydroxycinnamic acids loading positively on PC1, together with DPPH activity. In contrast, the water extract was positioned in the negative PC1 region, together with choline chloride:glucose (2:1) (NADES 1), choline chloride:xylose (2:1) (NADES 2), and choline chloride:glycerol (NADES 6), indicating a weaker association with the major phenolic compounds driving PC1 separation. Choline chloride:citric acid (1:2) (NADES 5) was clearly separated from the other extracts in the negative PC1 and positive PC2 region, suggesting a distinct solvent-dependent phenolic profile. PCA confirmed that solvent type strongly influenced both the phenolic composition and antioxidant characteristics of garlic peel waste extracts.

**Figure 10:**
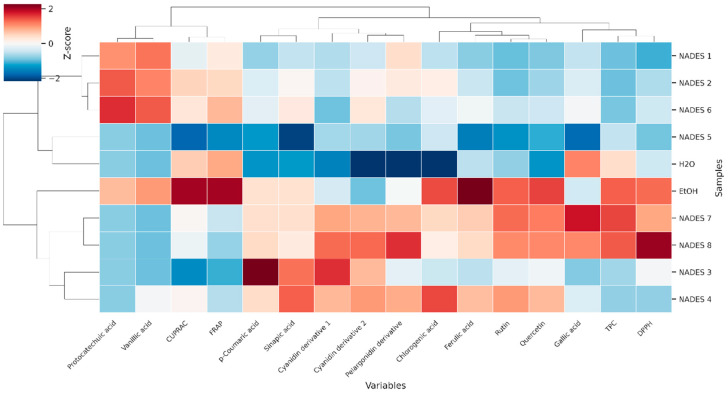
Hierarchical cluster analysis (HCA) map of the phenolic composition and antioxidant properties of garlic peel waste extracts.

HCA was performed to provide a more detailed evaluation of the similarities and differences among garlic peel waste extracts based on their phenolic composition and antioxidant properties (Figure 10). The extracts were grouped into two main clusters. The first cluster included NADES 1, NADES 2, NADES 6, NADES 5, and the water extract. Within this cluster, NADES 1, NADES 2, and NADES 6 exhibited relatively similar profiles and were characterized by lower levels of certain phenolic compounds and antioxidant parameters. The inclusion of the water extract and NADES 5 in the same broad cluster indicates that these extracts were less strongly associated with the phenolic-rich profiles observed in the other samples. This finding suggests that these solvents may have shown relatively lower efficiency in the extraction or preservation of the main phenolic compounds contributing to sample discrimination. The second main cluster consisted of choline chloride:malic acid (1:2) (NADES 3), NADES 4, NADES 7, NADES 8, and the ethanol extract. This grouping indicates that these extracts shared more similar profiles and were characterized by higher levels of phenolic compounds and stronger antioxidant properties. In particular, the close clustering of NADES 7 and NADES 8, together with their association with TPC, DPPH activity, gallic acid, rutin, quercetin, and various hydroxycinnamic acids, supports the effectiveness of lactic acid-containing NADES formulations in obtaining phenolic-rich garlic peel extracts. The clustering of NADES 3 and NADES 4 further suggests that these solvents may exhibit similar extraction selectivity for specific phenolic compounds, particularly certain anthocyanin derivatives and hydroxycinnamic acids. The positioning of the ethanol extract within the phenolic-rich cluster confirms its effectiveness as a conventional extraction solvent. However, the separation of the ethanol extract from NADES 7 and NADES 8 indicates that its profile was not identical to those of the most effective NADES extracts. The ethanol extract was more closely associated with higher CUPRAC and FRAP values, suggesting a stronger relationship with antioxidant responses based on reducing capacity. In comparison, NADES 7 and NADES 8 were more strongly associated with DPPH activity and with the flavonols, anthocyanins, and hydroxycinnamic acids that loaded positively on PC1. The clustering of variables further supported these findings. TPC, DPPH activity, and gallic acid were grouped closely together, while CUPRAC and FRAP formed a separate cluster. This pattern indicates that different antioxidant assays reflect distinct aspects of the antioxidant response. The HCA results confirmed that the extraction solvent had a decisive influence on the phenolic composition and antioxidant properties of garlic peel waste extracts. They also demonstrated that selected NADES formulations, NADES 7 and NADES 8, may exhibit profiles that are either comparable to or distinct from ethanol, depending on the antioxidant mechanism considered.

## 3. Materials and Methods

### 3.1. Material

In this study, Taşköprü garlic (*Allium sativum* L.) samples were used as the plant material and were purchased in triplicate from a local market. The garlic peels were manually separated, cleaned, and dried (UN55, Memmert GmbH Co. KG, Schwabach, Germany) at 30 °C until a constant weight was achieved prior to homogenization. The dried peels were then milled (Bosch, Munich, Germany) into a fine powder and sieved (Retsch GmbH, Haan, Germany) to obtain a homogeneous sample fraction (<1 mm). The resulting powdered material was stored at −20 °C until analysis.

### 3.2. Chemicals

Choline chloride (≥98%), used as the hydrogen bond acceptor (HBA) for the preparation of NADES, was purchased from Sigma-Aldrich (Steinheim, Germany). Lactic acid (≥90.0%) and glucose (≥97.5%), used as hydrogen bond donors (HBDs), were obtained from Isolab (Eschau, Germany), while glycerol (≥99.5%) was purchased from Supelco (Darmstadt, Germany). Xylose (≥98.0%), citric acid (≥98.0%), and malic acid (≥99.0%) were supplied by TCI (Tokyo, Japan). Prior to use, choline chloride was dried overnight at 100 °C.

The NADES formulations and their molar ratios were selected from solvent systems frequently used for the recovery of phenolic compounds from agri-food by-products [25,31,63,64,65,66,67,68,69,70] (Table 1). To represent a broad range of solvent compositions, hydrogen-bond donors from different chemical classes were included, comprising organic acids, sugars, and a polyol. Most were combined with choline chloride as the hydrogen-bond acceptor, while glycerol:lactic acid and glucose:lactic acid were included as choline chloride-free systems. This selection enabled the comparative evaluation of NADES with different chemical characteristics and their influence on the extraction of phenolic compounds from garlic peel waste.

For this purpose, the corresponding components were mixed at 80 °C using a magnetic stirrer (Dlab, Beijing, China) until a homogeneous and clear liquid was obtained. Subsequently, water was added to the mixtures at a concentration of 30% (*v*/*v*) to reduce viscosity, followed by vortexing (Four E’s Scientific, Guangzhou, China). To ensure complete dissolution, the mixtures were treated in an ultrasonic bath (DZG Science, Ankara, Türkiye) for 10 min. A 70% (*v*/*v*) ethanol solution and distilled water were used as reference solvents.

### 3.3. Determination of the Physical Properties of Natural Deep Eutectic Solvents (NADES)

#### 3.3.1. Density and pH

To determine the density of the NADES, 1 mL of each solvent sample was weighed at room temperature using an analytical balance (Mettler Toledo, Greifensee, Switzerland). The density of the solutions was calculated according to the equation ρ = m_NADES_/V_NADES_, where ρ represents the density, m_NADES_ refers to the mass of the weighed NADES sample, and V_NADES_ denotes the sample volume. The pH values of the NADES were measured using a pH meter (Ohaus ST400pH-G, Shanghai, China).

#### 3.3.2. Fourier Transform Infrared Spectroscopy (FTIR)

FTIR analysis was performed to characterize the principal functional groups and hydrogen-bonding environments of the prepared NADES and to identify formulation-dependent spectral differences associated with the use of sugars, polyols, and organic acids as solvent components. This analysis complemented the density and pH measurements by providing additional information on the chemical characteristics of the different solvent systems. FTIR spectra of the NADES were recorded at room temperature using a Shimadzu IRTracer-100 spectrometer (Kyoto, Japan). Measurements were performed over the wavenumber range of 4500–500 cm^−1^ with a resolution of 4 cm^−1^ and 32 scans per spectrum.

### 3.4. Ultrasound-Assisted Extraction of Phenolic Compounds and Storage Stability

The extraction of phenolic compounds was carried out based on a previously reported method [71]. Briefly, 10 mL of each NADES solution, prepared according to the molar ratios given in Table 1, was added to 0.5 g of garlic peel powder. The mixture was subjected to extraction for 30 min in an ultrasonic bath operating at 60 °C and 50 Hz (DZG Science, Ankara, Türkiye). After extraction, the mixture was centrifuged at 4500× *g* for 10 min using a refrigerated centrifuge (Sigma 3K30, Sigma Laborzentrifugen GmbH, Osterode am Harz, Germany), and the supernatant was transferred into a clean tube. The same procedure was also performed using 70% (*v*/*v*) ethanol and purified water as control solvents.

All prepared extracts were stored under two different temperature conditions (4 °C and 25 °C) for 60 days. During storage, samples were collected at 15-day intervals to monitor changes in total phenolic content and total antioxidant capacity.

### 3.5. In Vitro Gastrointestinal Digestion Simulation

The bioaccessibility of phenolic compounds was evaluated using the INFOGEST in vitro gastrointestinal digestion model, which simulates the gastric and intestinal phases of human digestion [27]. The gastric and intestinal electrolyte solutions used in the digestion procedure were prepared according to the compositions described in the cited INFOGEST method. Pepsin (P7012), pancreatin (P7545), and bile extract (B8631) were purchased from Sigma-Aldrich (Steinheim, Germany).

For the simulation of the gastric phase, 3.75 mL of gastric electrolyte solution, 0.8 mL of pepsin solution (25,000 U/mL), and 2.5 µL of CaCl_2_ solution (0.3 M) were added to 5 mL of extract. The pH of the mixture was adjusted to 3.0 using 1 M HCl, and the final volume was brought to 10 mL with distilled water. The mixture was then incubated in a shaking water bath (Memmert GmbH Co. KG, Schwabach, Germany) at 37 °C for 2 h.

For the simulation of the intestinal phase, 5.5 mL of intestinal electrolyte solution, 2.5 mL of pancreatin solution (800 U/mL), 1.25 mL of bile solution (160 mM), and 20 µL of CaCl_2_ solution (0.3 M) were added to the gastric digesta. The pH was adjusted to 7.0 using 1 M NaOH, and the final volume was brought to 20 mL with distilled water. The mixture was subsequently incubated in a shaking water bath at 37 °C for 2 h.

A blank sample was also prepared by applying the same digestion procedure in the absence of extract and was used to correct for potential interferences originating from the digestion fluids. Following the gastric and intestinal digestion phases, the collected samples were centrifuged, and the resulting supernatants were stored at −20 °C until analysis.

Bioaccessibility was calculated by dividing the amount of the compound determined after digestion by the amount determined before digestion and multiplying the resulting value by 100.

### 3.6. Determination of Total Phenolic Content

The total phenolic content was determined according to the method reported by Velioglu et al. [72]. Briefly, 0.75 mL of Folin–Ciocalteu reagent was added to 100 µL of extract, and the mixture was allowed to react for 5 min. Subsequently, 0.75 mL of 6% Na_2_CO_3_ solution was added, and the mixture was incubated at room temperature for 90 min. Before absorbance measurements, all samples were centrifuged at 4500× *g* for 5 min to eliminate insoluble particles. Absorbance readings were then measured at 725 nm using a spectrophotometer (Shimadzu UV-1800, Kyoto, Japan), against the corresponding NADES blanks to account for matrix effects. The total phenolic content of the extracts was calculated using a gallic acid calibration curve and the results were expressed as mg gallic acid equivalents (GAE)/100 g of garlic peel waste.

### 3.7. Determination of Total Antioxidant Capacity

#### 3.7.1. CUPRAC (Cupric Ion Reducing Antioxidant Capacity)

The CUPRAC assay was performed according to the method described by Apak et al. [73]. Briefly, 1 mL of copper (II) chloride solution (10 mM), 1 mL of neocuproine solution (7.5 mM), 1 mL of ammonium acetate buffer (1 M), and 1 mL of distilled water were sequentially added to 100 µL of extract, resulting in a final volume of 4.1 mL. The mixture was incubated at room temperature for 30 min, after which the absorbance was measured at 450 nm using a spectrophotometer (Shimadzu UV-1800, Kyoto, Japan), against the corresponding NADES blanks to account for matrix effects. The results were calculated using a Trolox^®^ calibration curve and expressed as mg Trolox^®^ equivalents (TE)/100 g of garlic peel waste.

#### 3.7.2. DPPH (2,2-Diphenyl-1-picrylhydrazyl) Radical Scavenging Activity

The DPPH assay was performed according to the method reported by Kumaran and Karunakaran [74]. Briefly, 2 mL of DPPH solution (0.1 mM) prepared in methanol was added to 100 µL of extract. The mixture was incubated in the dark for 30 min, after which the absorbance was measured at 517 nm using a spectrophotometer (Shimadzu UV-1800, Kyoto, Japan), against the corresponding NADES blanks to account for matrix effects. The results were calculated using a Trolox^®^ calibration curve and expressed as mg Trolox^®^ equivalents (TE)/100 g of garlic peel waste.

#### 3.7.3. FRAP (Ferric Reducing Antioxidant Power)

The FRAP assay was performed according to the method reported by Benzie and Strain [75]. Briefly, 900 µL of freshly prepared FRAP reagent was added to 100 µL of extract. The FRAP reagent was prepared by mixing acetate buffer (pH 3.6), 2,4,6-tripyridyl-*s*-triazine (TPTZ) solution (10 mM), and ferric chloride solution (20 mM) at a ratio of 10:1:1. After the addition of the reagent, the mixture was incubated at room temperature for 4 min, and the absorbance was measured at 593 nm using a spectrophotometer (Shimadzu UV-1800, Kyoto, Japan), against the corresponding NADES blanks to account for matrix effects. The total antioxidant capacity was calculated using a Trolox^®^ calibration curve, and the results were expressed as mg Trolox^®^ equivalents (TE)/100 g of garlic peel waste.

### 3.8. Quantification of Phenolic Compounds by HPLC–PDA

Quantitative analysis of phenolic compounds was performed using an HPLC–PDA system (Waters e2695, Milford, MA, USA) according to the method described by Kamiloglu et al. [76]. Chromatographic separation was achieved on a reversed-phase C18 column (4.6 mm × 250 mm, 5 µm). Prior to analysis, the samples were passed through a 0.45 µm membrane filter, and 10 µL of the filtered sample was injected into the system. Eluent A [trifluoroacetic acid in water (1:1000, *v*/*v*)] and eluent B [trifluoroacetic acid in acetonitrile (1:1000, *v*/*v*)] were used as the mobile phases, and the flow rate was set at 1.0 mL/min. The linear gradient elution program was as follows: 5% B at the beginning, a linear increase to 35% B from 0 to 45 min, an increase to 75% B from 45 to 47 min, a decrease to 35% B from 47 to 49 min, and a return to the initial conditions at 50 min. Spectral data were recorded at 280, 312, 360, and 520 nm. Rutin, quercetin, gallic acid, protocatechuic acid, vanillic acid, chlorogenic acid, *p*-coumaric acid, ferulic acid, and sinapic acid were identified and quantified by comparison of their retention times and UV–Vis spectral characteristics with those of the corresponding authentic standards. Two cyanidin derivatives and one pelargonidin derivative were tentatively identified based on their UV–Vis spectral characteristics and comparison with literature data. The cyanidin derivatives were quantified using a cyanidin 3-*O*-glucoside calibration curve, whereas the pelargonidin derivative was quantified using a pelargonidin 3-*O*-glucoside calibration curve. Calibration curves were constructed for all authentic standards over the concentration range of 0.1–200 µg/mL. The compound-specific limits of detection (LOD) and limits of quantification (LOQ), together with the calibration parameters, are presented in Supplementary Material, Table S2. The LOD values ranged from 2.01 to 21.63 µg/mL, while the LOQ values ranged from 6.10 to 65.54 µg/mL. The coefficients of determination (R^2^) ranged from 0.9918 to 0.9999. The results were expressed as µg/100 g of garlic peel waste.

### 3.9. Statistical Analysis

All analyses were performed in triplicate, and the results were expressed as mean ± standard deviation. Data were subjected to one-way analysis of variance (ANOVA) using IBM SPSS Statistics, version 29.0 (IBM Corp., Armonk, NY, USA). Significant differences among samples were determined using Tukey’s test (*p* < 0.05). Correlation analysis, principal component analysis (PCA), and hierarchical cluster analysis (HCA) were performed using Minitab Statistical Software, version 21.0 (Minitab LLC, State College, PA, USA).

## 4. Conclusions

This study showed that selected NADES formulations can support the recovery of phenolic compounds from garlic peel waste using UAE. However, their performance depended on solvent composition, target phenolic class, storage and digestion conditions, and the analytical endpoint evaluated. Glycerol:lactic acid (1:3) and glucose:lactic acid (1:5) produced the highest TPC values among the NADES formulations, whereas ethanol exhibited the strongest reducing capacity according to the CUPRAC and FRAP assays. HPLC–PDA analysis confirmed that extraction performance was compound-dependent. Ethanol was particularly effective for flavonols and hydroxycinnamic acids, while selected NADES formulations favored anthocyanin recovery and the post-digestion retention of flavonols and hydroxycinnamic acids. Chemometric analysis further showed that sample differentiation reflected the combined pattern of individual phenolic compounds and assay-specific antioxidant responses. Taken together, selected NADES formulations, particularly lactic acid-containing formulations, may represent promising alternatives to conventional solvents when selected according to the intended application and target phenolic class.

The findings should nevertheless be interpreted within the limitations of the study. Apparent bioaccessibility values derived from the TPC and antioxidant assays reflect changes in assay responses rather than quantitative compound recovery, while the in vitro digestion results should not be interpreted as evidence of in vivo absorption or bioavailability. In addition, compound-specific phenolic stability during storage could not be established because HPLC–PDA profiling was not performed at the storage intervals.

From a practical perspective, selected garlic peel-derived NADES extracts could potentially be developed as phenolic-rich ingredients or natural antioxidant systems for specific food applications, including beverages, sauces, bakery products, and edible coatings. Their production could also support the integration of garlic-processing waste into circular food production systems while reducing reliance on conventional organic solvents. In some applications, the possibility of incorporating the NADES extract directly into the final product without complete solvent removal may offer an additional technological advantage. However, industrial implementation requires further evaluation of extraction scale-up, solvent preparation and reuse, viscosity management, energy demand, process economics, and compatibility with existing processing lines. The effects of the extracts on product quality, sensory properties, stability, safety, and regulatory compliance must also be established in relevant food matrices. Encapsulation or carrier-based approaches may additionally improve their handling, long-term stability, controlled release, and suitability for specific applications.

## Figures and Tables

**Table 1:** Density and pH of the prepared NADES formulations.

NADES ^1^	HBA	HBD	Molar Ratio	Density (g/cm^3^)	pH
1	Choline chloride	Glucose	2:1	1.1721 ± 0.0071 ^bc^	3.46 ± 0.09 ^c^
2	Choline chloride	Xylose	2:1	1.1439 ± 0.0402 ^bc^	4.72 ± 0.06 ^a^
3	Choline chloride	Malic acid	1:2	1.2136 ± 0.0491 ^b^	0.53 ± 0.13 ^e^
4	Choline chloride	Lactic acid	1:2	1.1169 ± 0.0216 ^c^	1.23 ± 0.08 ^d^
5	Choline chloride	Citric acid	1:2	1.2047 ± 0.0143 ^b^	0.45 ± 0.04 ^e^
6	Choline chloride	Glycerol	1:2	1.3902 ± 0.0257 ^a^	4.10 ± 0.13 ^b^
7	Glycerol	Lactic acid	1:3	1.1777 ± 0.0047 ^bc^	1.25 ± 0.09 ^d^
8	Glucose	Lactic acid	1:5	1.1423 ± 0.0277 ^bc^	1.06 ± 0.14 ^d^

^1^ All solvents were diluted with 30% (*v*/*v*) water prior to analysis. Results are expressed as the mean of three measurements. Different letters within the columns represent statistically significant differences, as determined by one-way ANOVA followed by Tukey’s test (*p* < 0.05). HBA: hydrogen bond acceptor; HBD: hydrogen bond donor; NADES: natural deep eutectic solvent.

**Table 2:** Changes in the flavonoids of the garlic peel waste extracts during in vitro digestion ^1^.

	NADES 1	NADES 2	NADES 3	NADES 4	NADES 5	NADES 6	NADES 7	NADES 8	Ethanol	Water
Flavonols (µg/100 g of Garlic Peel Waste)										
Rutin										
Undigested	333.6 ± 32.8 ^f^	344.5 ± 35.4 ^f^	618.8 ± 24.5 ^d^	1050.8 ± 15.1 ^c^	179.0 ± 1.1 ^g^	511.9 ± 9.5 ^e^	1165.7 ± 21.8 ^ab^	1092.1 ± 6.3 ^bc^	1192.7 ± 26.7 ^a^	403.6 ± 9.1 ^f^
Digested	315.8 ± 42.7 ^c^	357.2 ± 13.0 ^bc^	694.2 ± 100.4 ^ab^	735.6 ± 146.5 ^a^	<LOQ	365.6 ± 15.3 ^bc^	696.4 ± 146.5 ^ab^	508.2 ± 37.1 ^abc^	<LOQ	<LOQ
Quercetin										
Undigested	479.9 ± 5.9 ^g^	631.8 ± 91.6 ^f^	1182.8 ± 55.7 ^d^	1909.2 ± 11.3 ^c^	238.2 ± 1.7 ^h^	886.2 ± 6.4 ^e^	2312.3 ± 16.7 ^b^	2226.8 ± 30.2 ^b^	2621.4 ± 11.6 ^a^	41.0 ± 0.3 ^i^
Digested	258.4 ± 2.1 ^ef^	374.0 ± 13.6 ^e^	608.1 ± 88.1 ^d^	1136.9 ± 22.8 ^b^	104.1 ± 22.6 ^f^	531.8 ± 22.1 ^d^	1485.9 ± 22.4 ^a^	965.6 ± 70.3 ^c^	606.1 ± 21.6 ^d^	178.2 ± 19.1 ^f^
Total flavonols										
Undigested	813.4 ± 34.3 ^h^	976.3 ± 75.1 ^g^	1801.6 ± 62.7 ^e^	2960.0 ± 18.1 ^d^	417.3 ± 2.8 ^i^	1398.1 ± 15.9 ^f^	3478.0 ± 38.5 ^b^	3318.9 ± 23.9 ^c^	3814.2 ± 38.3 ^a^	444.6 ± 9.3 ^i^
Digested	574.2 ± 44.8 ^c^	731.2 ± 26.7 ^c^	1302.3 ± 188.5 ^b^	1872.5 ± 293.5 ^a^	104.1 ± 22.6 ^d^	897.4 ± 37.3 ^c^	2182.3 ± 225.5 ^a^	1473.8 ± 107.4 ^b^	606.1 ± 21.6 ^c^	178.2 ± 19.1 ^d^
Anthocyanins (µg/100 g of garlic peel waste)										
Cyanidin derivative 1										
Undigested	32.5 ± 10.3 ^b^	35.2 ± 9.0 ^b^	109.8 ± 5.1 ^a^	79.1 ± 19.7 ^a^	30.3 ± 1.0 ^b^	21.1 ± 0.7 ^b^	84.6 ± 15.8 ^a^	97.6 ± 3.1 ^a^	40.8 ± 11.3 ^b^	<LOQ
Digested	<LOQ	<LOQ	<LOQ	<LOQ	<LOQ	<LOQ	<LOQ	<LOQ	<LOQ	<LOQ
Cyanidin derivative 2										
Undigested	127.4 ± 28.7 ^def^	165.6 ± 31.4 ^bcd^	210.5 ± 26.9 ^ab^	228.2 ± 6.6 ^ab^	108.5 ± 2.9 ^ef^	178.3 ± 4.8 ^bc^	216.7 ± 5.9 ^ab^	252.6 ± 3.0 ^a^	91.4 ± 14.7 ^f^	<LOQ
Digested	<LOQ	<LOQ	<LOQ	<LOQ	<LOQ	<LOQ	<LOQ	<LOQ	<LOQ	<LOQ
Pelargonidin derivative										
Undigested	37.5 ± 2.4 ^abcd^	34.9 ± 9.6 ^bcd^	29.0 ± 3.1 ^bcd^	44.1 ± 3.4 ^ab^	18.9 ± 0.2 ^d^	23.5 ± 0.3 ^cd^	42.5 ± 3.9 ^abc^	55.4 ± 0.7 ^a^	31.4 ± 14.5 ^bcd^	<LOQ
Digested	<LOQ	<LOQ	<LOQ	<LOQ	<LOQ	<LOQ	<LOQ	<LOQ	<LOQ	<LOQ
Total anthocyanins										
Undigested	197.4 ± 42.2 ^b^	235.7 ± 52.9 ^b^	349.3 ± 22.3 ^a^	351.5 ± 23.9 ^a^	157.7 ± 4.2 ^b^	222.9 ± 5.8 ^a^	343.8 ± 17.8 ^b^	405.6 ± 6.8 ^a^	163.6 ± 17.9 ^b^	<LOQ
Digested	<LOQ	<LOQ	<LOQ	<LOQ	<LOQ	<LOQ	<LOQ	<LOQ	<LOQ	<LOQ

^1^ Different letters within the rows represent statistically significant differences, as determined by one-way ANOVA followed by Tukey’s test (*p* < 0.05). LOQ: limit of quantification. The cyanidin derivatives were quantified as cyanidin-3-glucoside equivalents, whereas the pelargonidin derivative was quantified as pelargonidin-3-glucoside equivalents.

**Table 3:** Changes in the phenolic acids of the garlic peel waste extracts during in vitro digestion ^1^.

	NADES 1	NADES 2	NADES 3	NADES 4	NADES 5	NADES 6	NADES 7	NADES 8	Ethanol	Water
Hydroxybenzoic acids (µg/100 g of garlic peel waste)										
Gallic acid										
Undigested	1079.5 ± 18.9 ^b^	1233.3 ± 190.9 ^b^	770.5 ± 101.5 ^b^	1251.4 ± 11.4 ^b^	<LOQ	1447.3 ± 272.8 ^b^	3066.9 ± 578.0 ^a^	2433.3 ± 300.6 ^a^	1185.9 ± 223.5 ^b^	2467.9 ± 465.2 ^a^
Digested	<LOQ	<LOQ	<LOQ	<LOQ	<LOQ	<LOQ	671.0 ± 123.3 ^a^	530.3 ± 97.4 ^a^	<LOQ	<LOQ
Protocatechuic acid										
Undigested	592.2 ± 169.4 ^ab^	710.7 ± 123.4 ^ab^	<LOQ	<LOQ	<LOQ	799.5 ± 13.4 ^a^	<LOQ	<LOQ	494.1 ± 93.1 ^b^	<LOQ
Digested	<LOQ	<LOQ	<LOQ	<LOQ	<LOQ	<LOQ	<LOQ	<LOQ	<LOQ	<LOQ
Vanillic acid										
Undigested	1784.6 ± 6.4 ^a^	1688.9 ± 255.8 ^a^	<LOQ	723.1 ± 182.0 ^b^	<LOQ	1913.9 ± 32.0 ^a^	<LOQ	<LOQ	1563.2 ± 26.2 ^a^	<LOQ
Digested	<LOQ	<LOQ	<LOQ	<LOQ	<LOQ	<LOQ	<LOQ	<LOQ	<LOQ	<LOQ
Total hydroxybenzoic acids										
Undigested	3456.4 ± 175.0 ^ab^	3632.9 ± 516.9 ^a^	770.5 ± 101.5 ^d^	1974.5 ± 177.9 ^c^	<LOQ	4160.8 ± 318.1 ^a^	3066.9 ± 578.0 ^abc^	2433.3 ± 300.6 ^bc^	3243.3 ± 342.8 ^ab^	2467.9 ± 465.2 ^bc^
Digested	<LOQ	<LOQ	<LOQ	<LOQ	<LOQ	<LOQ	671.0 ± 123.3 ^a^	530.3 ± 97.4 ^a^	<LOQ	<LOQ
Hydroxycinnamic acids (µg/100 g of garlic peel waste)										
Chlorogenic acid										
Undigested	654.8 ± 7.8 ^d^	872.1 ± 125.6 ^bc^	680.1 ± 21.8 ^d^	1285.3 ± 37.3 ^a^	687.6 ± 6.4 ^d^	746.4 ± 6.9 ^cd^	962.9 ± 8.9 ^b^	870.3 ± 24.1 ^bc^	1278.4 ± 26.3 ^a^	138.6 ± 2.9 ^e^
Digested	<LOQ	<LOQ	674.1 ± 133.6 ^bc^	892.9 ± 199.3 ^ab^	<LOQ	428.3 ± 53.0 ^c^	1072.6 ± 100.4 ^a^	887.4 ± 136.9 ^ab^	<LOQ	<LOQ
*p*-Coumaric acid										
Undigested	252.4 ± 1.5 ^d^	343.7 ± 3.2 ^c^	837.2 ± 33.5 ^a^	483.0 ± 0.9 ^b^	138.5 ± 0.4 ^e^	357.9 ± 0.9 ^c^	473.2 ± 1.2 ^b^	485.0 ± 86.3 ^b^	468.2 ± 1.2 ^b^	131.3 ± 0.3 ^e^
Digested	<LOQ	160.0 ± 9.8 ^bcd^	261.1 ± 17.4 ^b^	451.4 ± 5.8 ^a^	102.8 ± 1.3 ^de^	136.8 ± 1.8 ^cd^	229.8 ± 50.3 ^bc^	424.0 ± 28.2 ^a^	451.9 ± 73.7 ^a^	243.7 ± 3.2 ^bc^
Ferulic acid										
Undigested	831.0 ± 3.0 ^f^	1136.3 ± 2.9 ^e^	1070.7 ± 17.9 ^e^	2157.2 ± 7.5 ^b^	145.5 ± 0.7 ^g^	1471.9 ± 12.9 ^d^	2043.3 ± 152.1 ^bc^	1935.8 ± 17.0 ^c^	3548.2 ± 53.1 ^a^	1067.8 ± 18.5 ^e^
Digested	266.5 ± 4.8 ^d^	410.6 ± 0.6 ^d^	464.6 ± 1.7 ^d^	1410.4 ± 52.9 ^ab^	<LOQ	1357.1 ± 95.0 ^b^	811.8 ± 11.1 ^c^	1435.3 ± 113.5 ^ab^	1719.1 ± 162.2 ^a^	838.2 ± 122.7 ^c^
Sinapic acid										
Undigested	503.9 ± 25.0 ^c^	625.7 ± 38.4 ^b^	905.4 ± 9.5 ^a^	928.4 ± 11.4 ^a^	135.7 ± 0.7 ^e^	670.3 ± 11.6 ^b^	703.7 ± 67.0 ^b^	667.5 ± 6.4 ^b^	699.8 ± 4.0 ^b^	312.7 ± 1.8 ^d^
Digested	<LOQ	<LOQ	<LOQ	<LOQ	<LOQ	<LOQ	<LOQ	<LOQ	<LOQ	<LOQ
Total hydroxycinnamic acids										
Undigested	2242.0 ± 18.4 ^f^	2977.8 ± 149.1 ^e^	3493.4 ± 69.5 ^d^	4854.0 ± 34.6 ^b^	1107.3 ± 5.3 ^h^	3246.5 ± 4.6 ^d^	4183.0 ± 77.4 ^c^	3958.6 ± 51.5 ^c^	5994.7 ± 76.6 ^a^	1650.3 ± 19.2 ^g^
Digested	266.5 ± 4.8 ^de^	570.6 ± 9.2 ^d^	1399.8 ± 152.7 ^c^	2754.7 ± 152.2 ^a^	102.8 ± 1.3 ^e^	1922.2 ± 43.7 ^b^	2114.3 ± 61.1 ^b^	2746.6 ± 51.6 ^a^	2171.1 ± 235.8 ^b^	1081.9 ± 125.8 ^c^

^1^ Different letters within the rows represent statistically significant differences, as determined by one-way ANOVA followed by Tukey’s test (*p* < 0.05). LOQ: limit of quantification.

## Data Availability

Dataset available on request from the authors.

## Supplementary Materials

The following supporting information can be downloaded at: https://www.mdpi.com/article/10.3390/molecules31142491/s1, Table S1: Variance ratios explained by the first three principal components; Table S2: Calibration characteristics, limits of detection, and limits of quantification of the phenolic standards used for HPLC–PDA analysis; Figure S1. Fourier transform infrared spectroscopy (FTIR) spectra of the prepared natural deep eutectic solvent formulations (NADES); Figure S2. Correlation between the phenolic composition and antioxidant properties of garlic peel waste extracts.

## Abbreviations

The following abbreviations are used in this manuscript:

CUPRAC	Cupric Ion Reducing Antioxidant Capacity
DPPH	2,2-Diphenyl-1-picrylhydrazyl
FRAP	Ferric Reducing Antioxidant Power
FTIR	Fourier Transform Infrared Spectroscopy
GAE	Gallic Acid Equivalents
HBA	Hydrogen Bond Acceptor
HBD	Hydrogen Bond Donor
HCA	Hierarchical Cluster Analysis
HPLC–PDA	High-Performance Liquid Chromatography–Photodiode Array Detection
LOD	Limit of Detection
LOQ	Limit of Quantification
NADES	Natural Deep Eutectic Solvent
PCA	Principal Component Analysis
TAC	Total Antioxidant Capacity
TE	Trolox Equivalents
TPC	Total Phenolic Content
UAE	Ultrasound-Assisted Extraction
