# Supplementary material for: Valorization of Garlic (Allium sativum L.) Peel Waste Using Natural Deep Eutectic Solvent (NADES) Formulations: Storage Stability and Bioaccessibility of Phenolic Extracts
Source: Molecules. 2026 Jul 16;31(14):2491. doi: 10.3390/molecules31142491 (PMC13414839; doi:10.3390/molecules31142491)
Supplement: Supplementary file 1 [file molecules-31-02491-s001.zip › molecules-4413197-supplementary.pdf]

Table S1. Variance ratios explained by the first three principal components

Variables	PC1	PC2	PC3
Eigenvalue	7.0189	3.9498	2.7360
Percent (%)	43.9	24.7	17.1
Cumulative	43.9	68.6	85.7
TPC	0.609	-0.205	0.725
CUPRAC	0.306	-0.927	0.089
DPPH	0.778	-0.127	0.436
FRAP	-0.011	-0.977	0.033
Cyanidin derivative 1	0.769	0.602	-0.124
Cyanidin derivative 2	0.675	0.471	-0.430
Pelargonidin derivative	0.759	0.179	-0.340
Rutin	0.952	-0.150	0.176
Quercetin	0.970	-0.140	0.035
Gallic acid	0.431	-0.187	0.589
Protocatechuic acid	-0.234	-0.669	-0.592
Vanillic acid	-0.144	-0.700	-0.672
Chlorogenic acid	0.725	-0.173	-0.440
p-Coumaric acid	0.707	0.319	-0.313
Ferulic acid	0.788	-0.561	0.053
Sinapic acid	0.756	0.031	-0.470

Table S2. Calibration characteristics, limits of detection, and limits of quantification of the phenolic standards used for HPLC–PDA analysis

Standard	LOD (µg/mL)	LOQ (µg/mL)	Slope	Intercept	R^2
Rutin	15.44	46.79	15,778	−26,840	0.9958
Quercetin	17.92	54.29	87,832	−202,192	0.9944
Cyanidin 3-*O*-glucoside	21.63	65.54	41,309	67,565	0.9918
Pelargonidin 3-*O*-glucoside	20.06	60.79	117,772	268,648	0.9929
Gallic acid	16.11	48.82	46,633	−146,481	0.9957
Protocatechuic acid	15.88	48.11	19,868	−60,629	0.9958
Vanillic acid	16.01	48.52	27,922	−84,761	0.9957
Chlorogenic acid	2.01	6.10	44,176	−14,530	0.9999
p-Coumaric acid	17.74	53.75	98,035	331,727	0.9945
Ferulic acid	16.43	49.79	65,080	204,743	0.9953
Sinapic acid	2.73	8.28	47,453	−37,887	0.9999

LOD, limit of detection; LOQ, limit of quantification. Calibration range: 0.1–200 µg/mL.

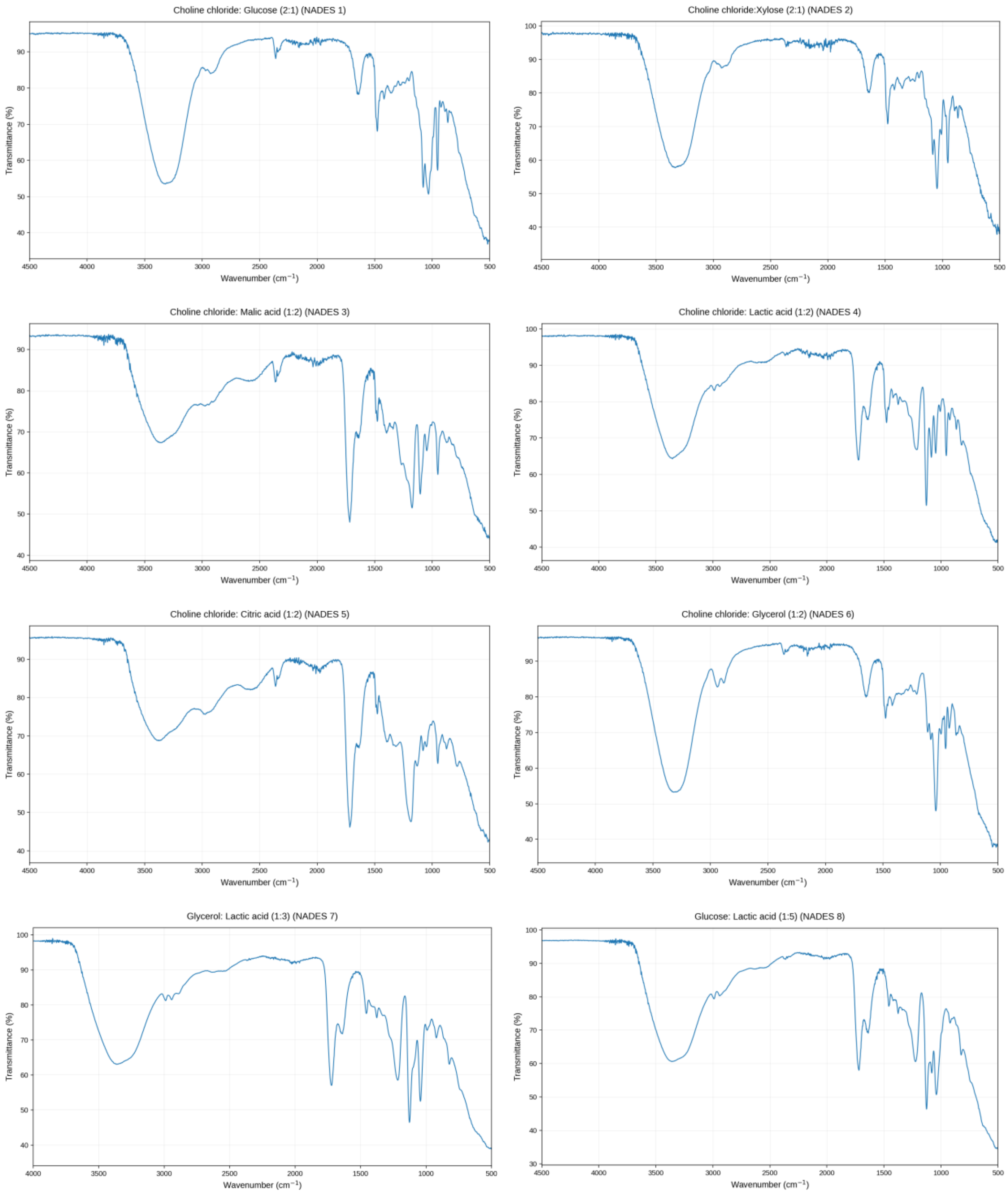

Figure S1. Fourier transform infrared spectroscopy (FTIR) spectra of the prepared natural deep eutectic solvents (NADES)

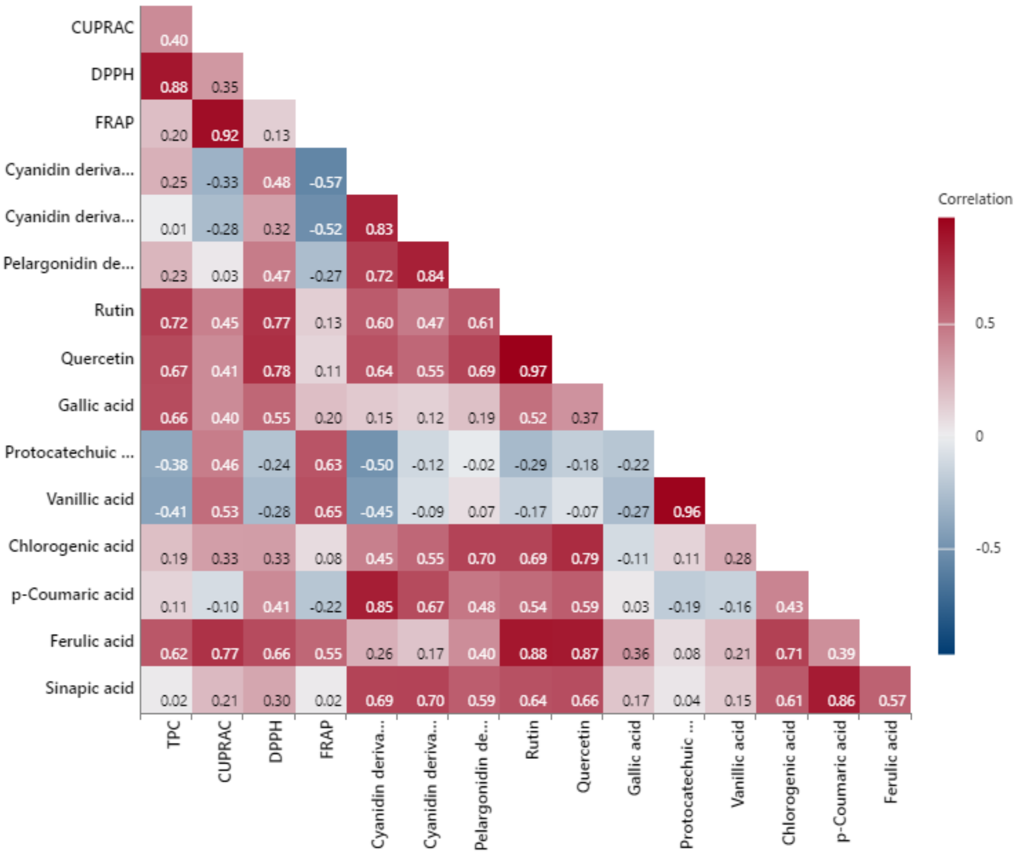

Figure S2. Correlation between the phenolic composition and antioxidant properties of garlic peel waste extracts.
